# Supplementary material for: Screening the Marker Components in Psoralea corylifolia L. with the Aids of Spectrum-Effect Relationship and Component Knock-Out by UPLC-MS2
Source: Int J Mol Sci. 2018 Nov 2;19(11):3439. doi: 10.3390/ijms19113439 (PMC6274892; doi:10.3390/ijms19113439)
Supplement: Supplementary file 1 [file ijms-19-03439-s001.pdf]

Screening the Marker Components in *Psoralea corylifolia* L. with the Aids of spectrum-effect relationship and Component Knock-out by UPLC-MS^2

Author names:
Changqin Li [1,2‡], Mengjun Shi [1,2‡], Junya Wang [1], Yong Sun [1,4], Yan Zhang [1,3], Wen-yi Kang [1,2*]

Author affiliations
[1] Joint International Research Laboratory of Food & Medicine Resource Function, Henan Province, Henan University, Kaifeng 475004, China.
[2] Institute of Chinese Materia Medica, Henan University, Kaifeng 475004, China.
[3] Hebei Food Inspection and Research Institute, Shijiazhuang 050091, China.
[4] Beijing Academy of Food Sciences, China Meat Research Center, Beijing 100068, China.
[‡]These authors contributed equally to this work.

Corresponding author details:

*Correspondence to: Wenyi Kang, Tel: ＋86 10-23880680; fax: ＋86 10-23880680; Email: kangweny@hotmail.com; Postal adress: Jinming Avenue, Henan University, Kaifeng 475004, China.

Table S1 The relative retention times and characteristic peak areas of each *P. corylifolia* sample measured by HPLC (1).

No.	P1	P2	P3	P4	P5	P6	P7	P8	P9	P10
Retention Time(min)	24.171	25.466	29.741	34.862	37.461	40.518	44.271	45.673	46.995	48.471
S1	1910171	1946360	1317112	898002.3	41593880	77056040	3010873	1610129	2210408	1882357
S2	4931025	7671755	1084838	2213777	96156350	50461660	1204003	5012484	4842989	722034.6
S3	1265623	1176642	936200.2	82215650	75796700	1545073	1573467	1770240	1107530	3085340
S4	2482634	2956066	2676640	87734540	84331340	5272716	2899254	3352454	3202544	5384064
S5	3497042	5263931	1481971	1.14E+08	1.14E+08	1897839	1806078	1473797	1774865	1580942
S6	7562726	4902941	4484689	98277600	89242290	3422701	7941749	8178495	4337410	3175305
S7	7240782	5687824	6062279	99660230	1.02E+08	4207686	3896773	2883527	3912045	3050735
S8	4924368	4476076	3130060	56281080	1.08E+08	2974597	3456537	4215263	2959213	4526070
S9	2144550	1999321	2054560	91964750	93945200	1945295	1338366	1704618	1315198	3806032
S10	2956203	2451960	2705462	1.14E+08	1.11E+08	1959457	2599175	2971888	2557256	6088901

Table S2 The relative retention times and characteristic peak areas of each *P. corylifolia* sample measured by HPLC (2).

No.	P11	P12	P13	P14	P15	P16	P17	P18	P19	P20
Retention Time(min)	54.265	59.614	61.75	63.619	67.106	69.798	70.977	72.94	75.433	77.379
S1	4387219	4924735	77377750	12354790	18344610	17456860	8426833	21531060	7042646	1027617
S2	936421	3513428	26386760	5583652	11352740	17011020	9334032	25181440	13037630	3602447
S3	3379895	68940130	10297280	13897690	16740930	9522331	16731780	4157447	2095937	29032940
S4	4723850	73570030	11330930	16897640	19127470	9443427	26192500	8199690	1665061	30760460
S5	4645381	5330264	78613030	9607765	15331270	23923060	13710360	45444070	12818310	8410219
S6	6946191	92636930	12434790	25530230	27100100	15797370	76998250	43766980	13756720	52134870
S7	5488332	6096722	40803080	14003520	15760150	24265490	13993270	59800820	17367990	8396749
S8	5308022	91049840	14932430	19488140	21013750	11978830	38433860	15863440	3033119	42666760
S9	3364813	73475760	10857930	12628730	16629700	7957881	21102260	4413539	999205.4	35904300
S10	5822505	90111810	15325430	26493170	23823150	14181130	25071530	8060044	2367130	36874970

Table S3 The relative retention times and characteristic peak areas of each *P. corylifolia* sample measured by HPLC (3).

No.	P21	P22	P23	P24	P25	P26	P27	P28	P29	P30
Retention Time(min)	80.354	84.743	88.176	91.43	95.749	98.433	99.824	102.073	106.201	108.495
S1	39070780	5936956	30717810	38252920	57609060	981166.8	57905280	1513077	3630110	1.05E+08
S2	7619369	1419304	10195570	6637759	16217200	1530603	23686470	438831.2	6195911	1.01E+08
S3	5112052	26418060	32185780	40499470	718601.8	24147030	3711368	1836312	35578350	87453720
S4	4357970	26483470	41617810	42317820	1221740	44067000	5952085	5885711	23675130	77847980
S5	44358340	6092755	22784000	46971360	62331360	1429803	43562710	7145450	12648380	9249037
S6	5926343	36882860	63438640	64272810	7381568	18679850	13590430	27690930	29660510	37184440
S7	35746020	5283760	29112520	47512900	66811130	5407201	47580130	11063190	21141930	22841850
S8	4700841	34157470	53081660	69312900	4253129	45309500	9527512	11786770	32708020	4390750
S9	2235243	23381550	38657060	44370440	2769637	53421100	2400802	3307228	36158160	14378870
S10	1997108	52999830	55009220	53508280	1888468	43842490	7982105	2259706	12192640	9453751

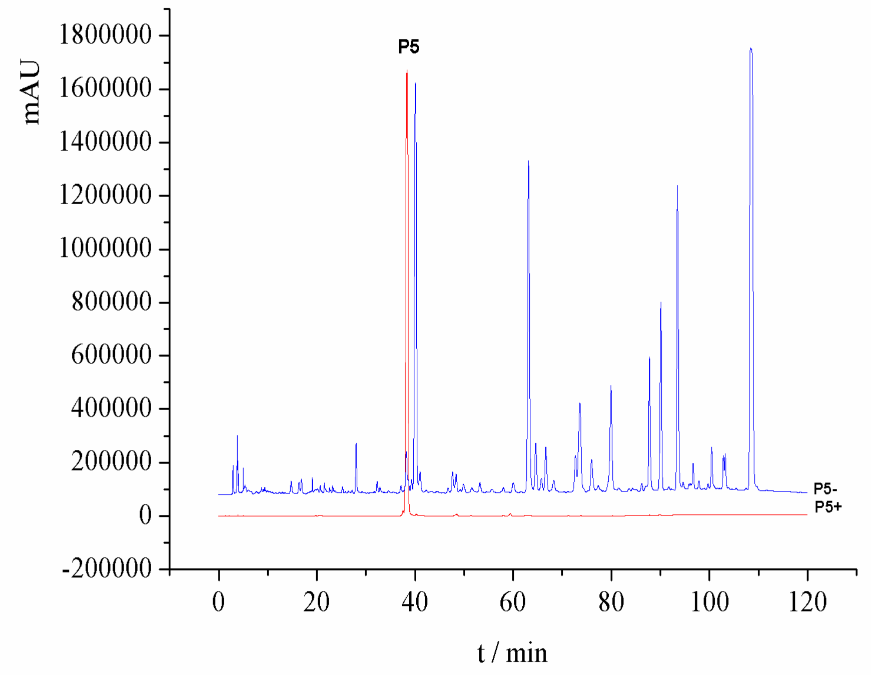
mAU
1800000
1600000
1400000
1200000
1000000
800000
600000
400000
200000
0
-200000
P5
P5-
P5+
0
20
40
60
80
100
120
t / min

(c)

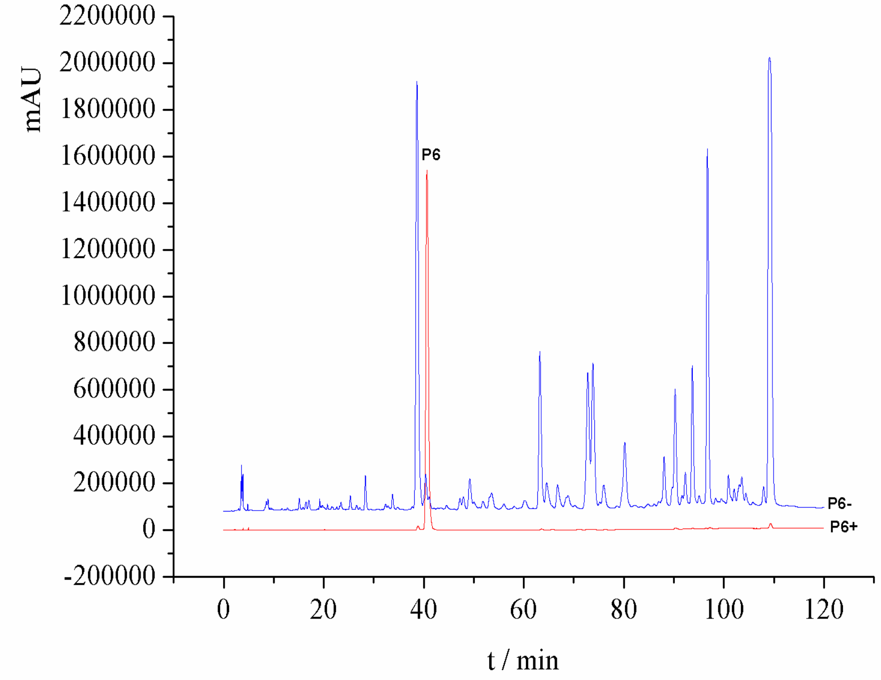
mAU
2200000
2000000
1800000
1600000
1400000
1200000
1000000
800000
600000
400000
200000
0
-200000
P6
P6-
P6+
0
20
40
60
80
100
120
t / min

(d)

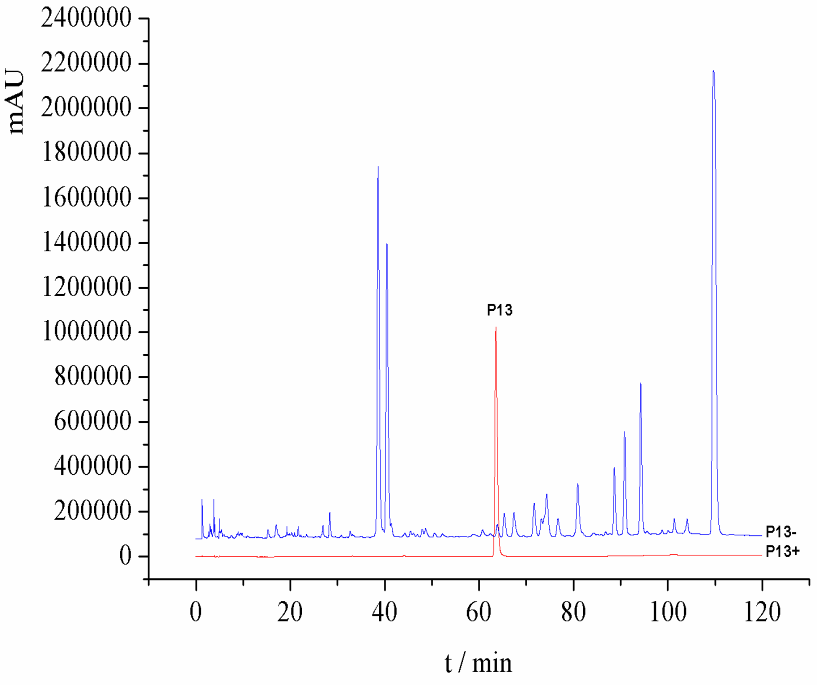

mAU
2400000
2200000
2000000
1800000
1600000
1400000
1200000
1000000
800000
600000
400000
200000
0
P13
P13-
P13+
0
20
40
60
80
100
120
t / min

(e)

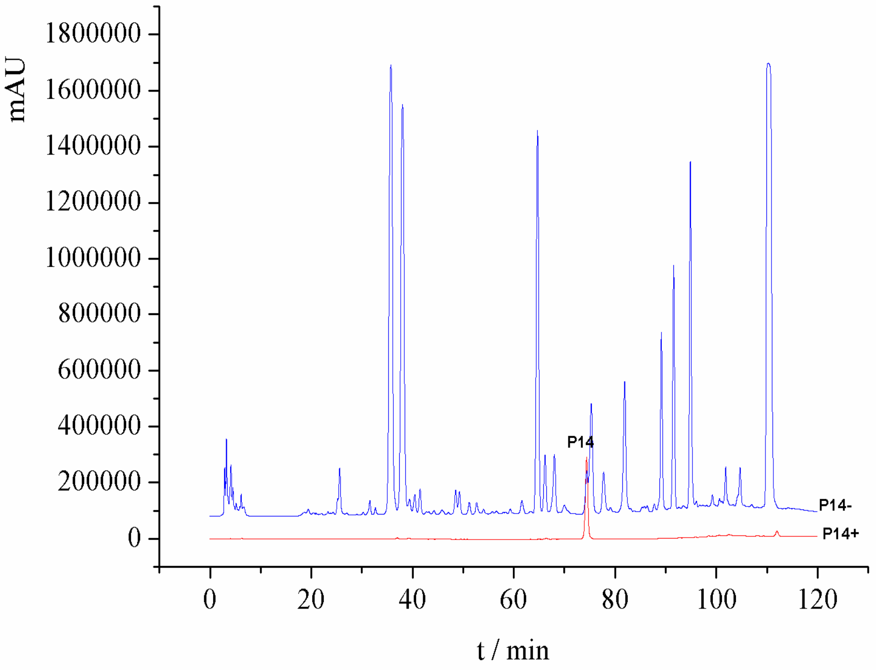

mAU
1800000
1600000
1400000
1200000
1000000
800000
600000
400000
200000
0
P14
P14-
P14+
0
20
40
60
80
100
120
t / min

(f)

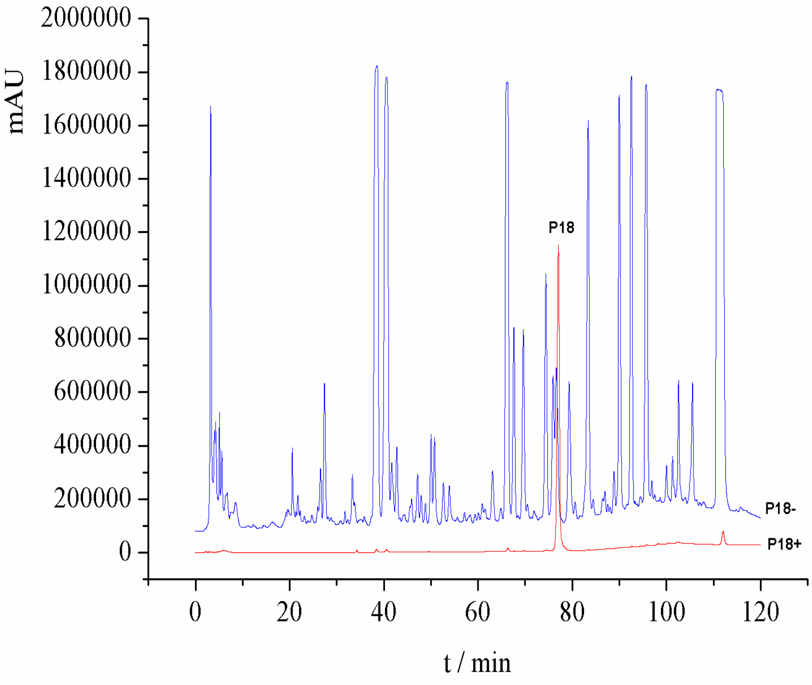

mAU
2000000
1800000
1600000
1400000
1200000
1000000
800000
600000
400000
200000
0
P18
P18-
P18+
0
20
40
60
80
100
120
t / min

(g)

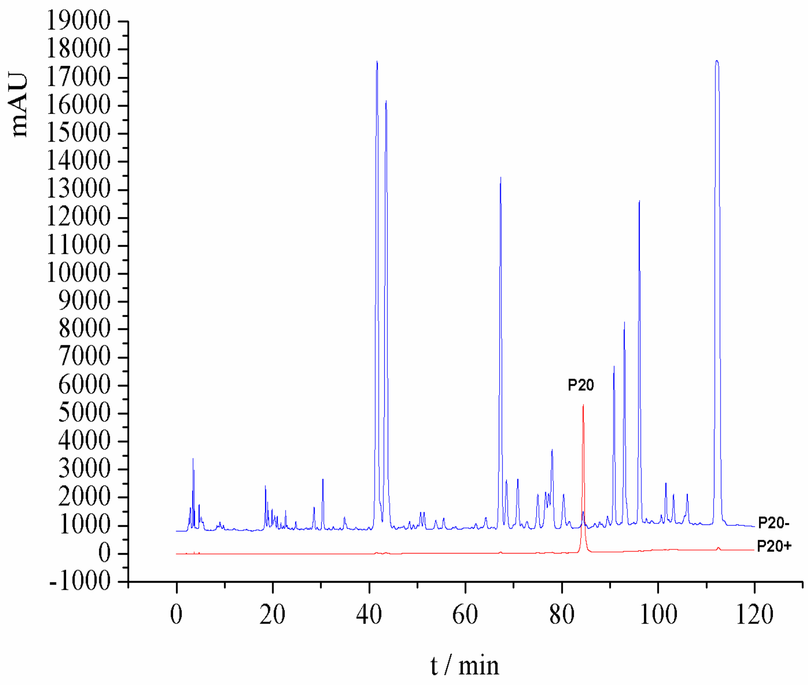

mAU
19000
18000
17000
16000
15000
14000
13000
12000
11000
10000
9000
8000
7000
6000
5000
4000
3000
2000
1000
0
-1000
P20
P20-
P20+
0
20
40
60
80
100
120
t / min

（**h**）

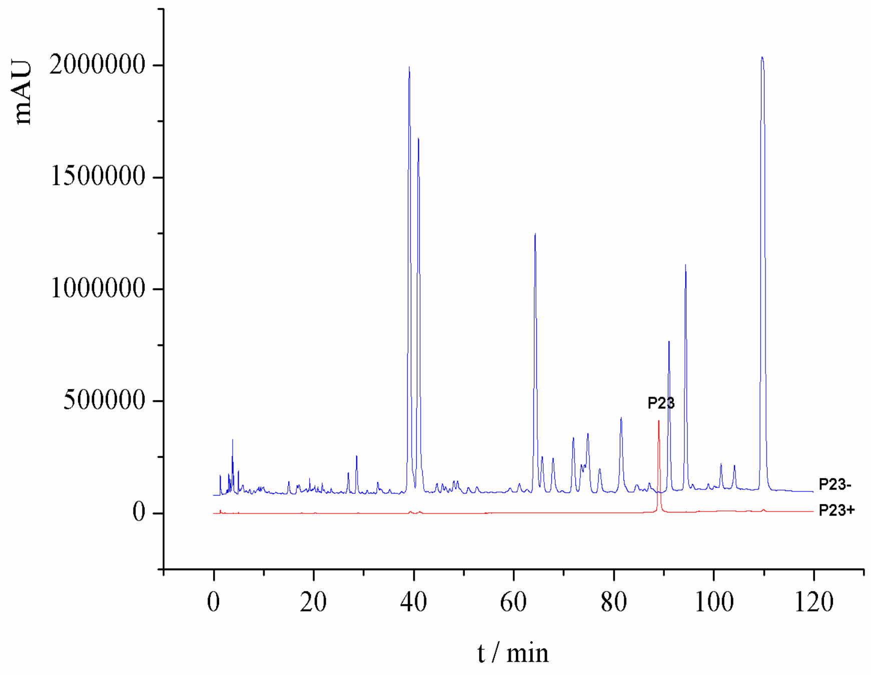
mAU
2000000
1500000
1000000
500000
0
0
20
40
60
80
100
120
t / min
P23
P23-
P23+

(i)

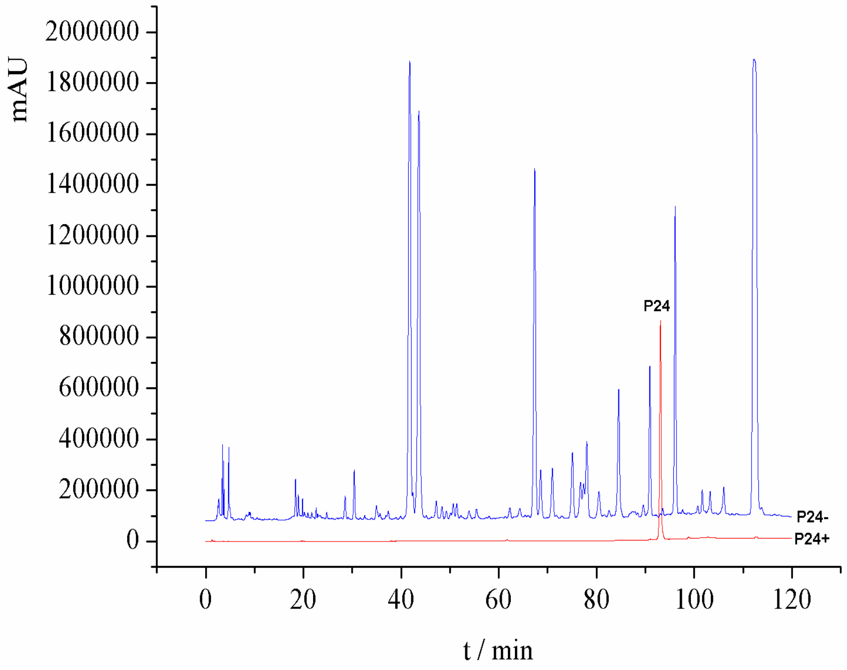
mAU
2000000
1800000
1600000
1400000
1200000
1000000
800000
600000
400000
200000
0
0
20
40
60
80
100
120
t / min
P24
P24-
P24+

(j)

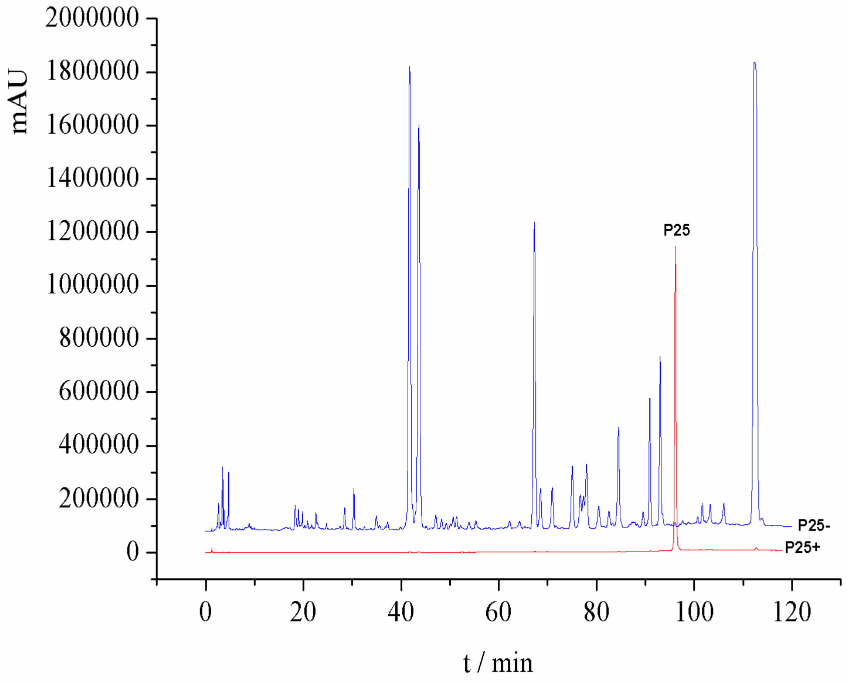

2000000
1800000
1600000
1400000
1200000
1000000
800000
600000
400000
200000
0
mAU
P25
P25-
P25+
0
20
40
60
80
100
120
t / min

(k)

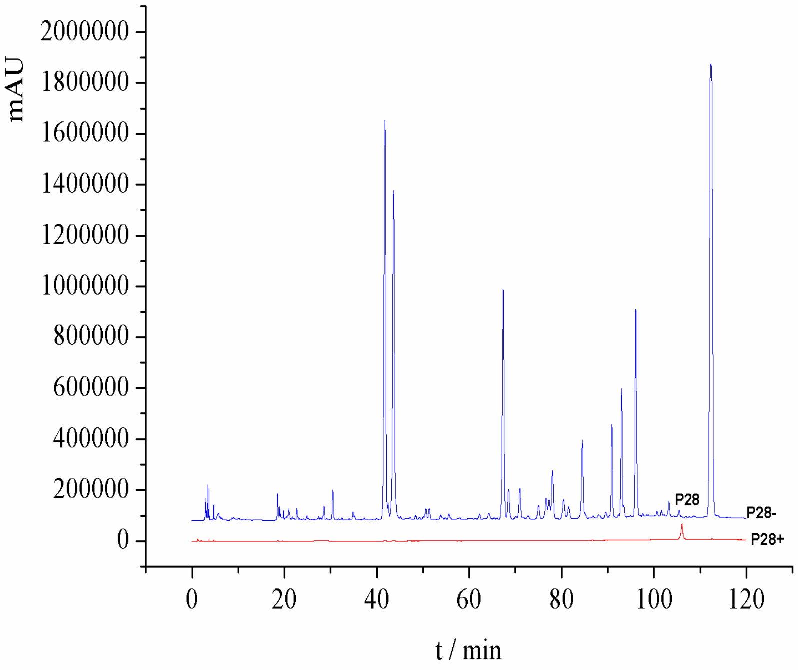

2000000
1800000
1600000
1400000
1200000
1000000
800000
600000
400000
200000
0
mAU
P28
P28-
P28+
0
20
40
60
80
100
120
t / min

(l)

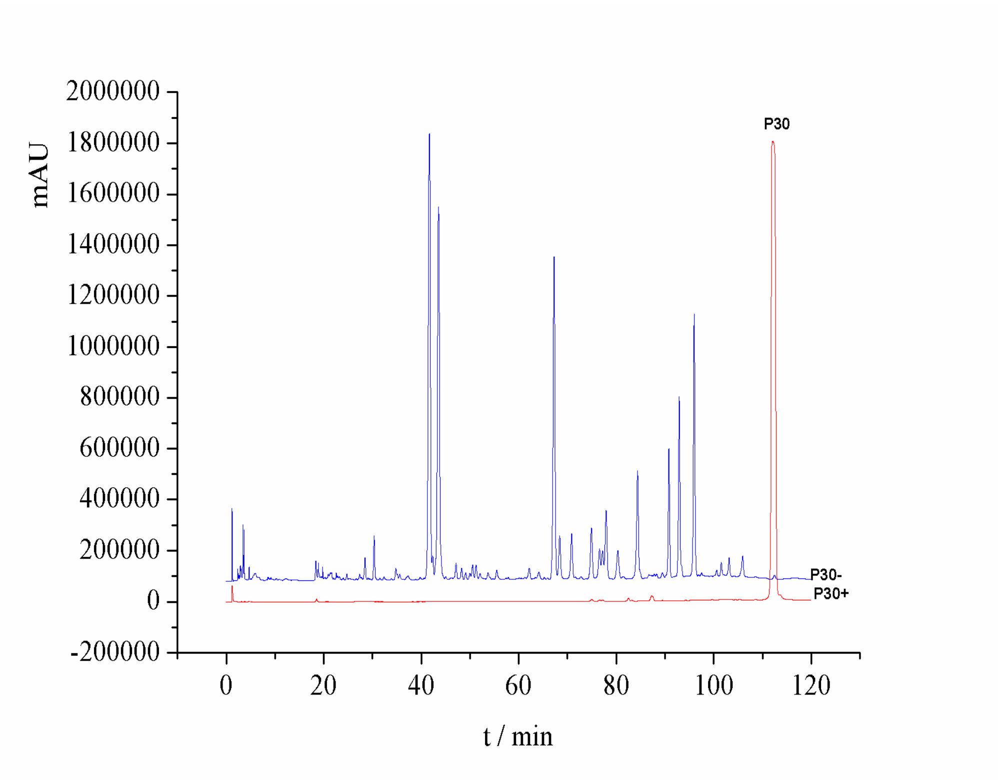

(m)

Figure S1 (c-m) A component (P_{X+}) and negative samples (P_{X-})
